# Carpal tunnel syndrome in patients with arteriovenous fistula for haemodialysis: A narrative review of the current literature

**DOI:** 10.1177/1129729820948690

**Published:** 2020-08-11

**Authors:** Yasmin Grant, Simon Freilich, Maxim D Horwitz, David Shemesh, Jeremy Crane

**Affiliations:** 1 Department of Transplant Surgery, Imperial College Renal and Transplant Centre, Hammersmith Hospital, London, UK; 2 Department of Clinical Neurophysiology, Luton and Dunstable University Hospital, NHS Foundation Trust, Luton, UK; 3 Department of Hand Surgery, Chelsea and Westminster Hospital NHS Trust, London, UK; 4 Department of Surgery and Haemodialysis Access Unit, Shaare Zedek Medical Centre, Jerusalem, Israel

**Keywords:** Arteriovenous fistula, carpal tunnel, carpal tunnel syndrome, vascular access, haemodialysis

## Abstract

The ideal choice of vascular access in patients requiring haemodialysis is an arteriovenous fistula. However, an important often under-reported complication encountered at follow-up is symptoms of tingling or numbness in the hand. This may represent carpal tunnel syndrome, impairment of the median nerve as it traverses through the carpal tunnel at the wrist by focal compression of this nerve. Contributory factors in the presence of an arteriovenous fistula may include venous hypertension and varying steal syndrome phenomena provoking micro-ischaemia. Studies that investigated the evolution of carpal tunnel syndrome in haemodialysis patients with an arteriovenous fistula revealed that the frequency of carpal tunnel syndrome associated with an arteriovenous fistula on haemodialysis ranged from 10.4% to 42.6%. An association between duration of haemodialysis with arteriovenous fistula and carpal tunnel syndrome development was also observed. Surgical release of carpal tunnel provided complete relief of paraesthesia in all treated patients in the examined, demonstrating an alleviation of symptoms and improved function of hand and quality of life in patients with an arteriovenous fistula. However, the aetiology and risk factors for development of carpal tunnel syndrome remain unclear and further studies should attempt to elucidate the pathophysiology of this occurrence in the presence of arteriovenous fistulas.

## Introduction

The ideal choice of vascular access in patients requiring haemodialysis (HD) is an arteriovenous fistula (AVF). The benefits of this include reduced infection rates with hospital re-admissions and prolonged survival compared with arteriovenous grafts and central venous catheters.^1^ However, there are longer term complications which include thrombosis, aneurysm formation and vascular steal phenomenon.^2^ An important short- to medium-term finding often encountered at follow up is symptoms of tingling or numbness in the hand. The symptoms are often suggestive of carpal tunnel syndrome (CTS),^3^ although, this may not always be the case. The purpose of this narrative review is to highlight clinical, neurophysiological and histopathological insights into the appropriate diagnosis and management of the tingling hand post-AVF formation.

CTS is the most common entrapment neuropathy affecting around 10% of the UK population with a prevalence of around 3%.^4–6^ It is caused by impairment of the median nerve as it traverses through the carpal tunnel at the wrist by focal compression of this nerve,^7,8^ as well as local ischaemia.^9^ While CTS can present acutely such as following a wrist fracture, it is most often due to a chronic build-up of multiple elements which lead to increased pressure within the carpal tunnel. Histopathological findings show thickening of the flexor tendons, oedema,^10^ collagen proliferation, fibrosis, amyloid deposition and thickening of blood vessel walls^11^ which all contribute to the above process.^12^ Those depositions are often precipitated by the well-known aetiological risk factors of chronic friction of the flexor tendons, or patients with tenosynovitis of the wrist flexor tendons.^12^

However, not all CTS is attributable to chronic tendon friction, as common risk factors include genetic predispositions (positive family history without occupational repetitive hand use), obesity, hypothyroidism, inflammatory arthropathy such as rheumatoid arthritis and diabetes.^13–15^ Patients with diabetes are at increased risk of macro- and micro-vascular complications which, of course, include end stage renal failure (ESRF) and requirement for HD. Therefore, the HD patient cohort (many of whom are diabetic) is at increased risk of having pre-existing CTS even before AVF formation.^16^

To complicate matters further, it has been shown experimentally that ischaemic provocation of patients with CTS via a tourniquet will worsen CTS symptoms as well as increase neurophysiological conduction abnormalities.^17^ Thus, the presence of an AVF (often associated with varying steal syndrome phenomena) could precipitate symptoms of a pre-existent asymptomatic carpal tunnel lesion via additional micro-ischaemia, as well as independently increasing inflammatory depositions within the carpal tunnel.^18^ A further theory regarding development of CTS in AVF formation includes venous hypertension leading to compression of the median nerve.^4,11,12^ Amyloidosis, specifically β2-microglobulin amyloidosis, is also a serious complication of long-term HD and hence, link duration of HD with CTS,^6,19^ independent of AVF formation.

It is clear that the pathophysiology of CTS in patients dialysing with AVFs is currently unknown and there is controversy as to whether AVF increases the risk of CTS developing. Correct and timely interventions are required for these different conditions and hence, the need to review the known literature to identify an evidence-based approach.

A narrative review of the literature revealed the following summarised in Table 1.

**Table 1.** Study descriptions and demographics.

Author	Year	Study type	Number of patients with CTS/total number of patients (%)	Site of AVF	Mean age in years (range)	M:F ratio	Diagnosis of CTS	Use of steroid therapy (n)	Mean length of HD
Harding and Fanu^20^	1997	Case series	2/2	Two ipsilateral	69 (42–54)	1:1	Clinical examination	Yes (1)	18 months
Khan^21^	2008	Observational	19/19	Undisclosed	59 (41–48)	12:7	NCS	No	12.6 years
Kimura et al.^22^	1986	Case series	16 (22 extremities)	Undisclosed	Undisclosed	Undisclosed	NCS	Yes (20)	Undisclosed
Kocyigit et al.^23^	2013	Observational	12/12	Undisclosed	63 ± 7	7/5	NCS	No	Undisclosed
Kopec et al.^24^	2010	Observational	40/386 (10.4%)	14 ipsilateral, 23 bilateral, 3 contralateral	54.5 (36–83)	2:1	NCS	No	16.05 months (18–100)
Kumar et al.^25^	1975	Case report	2/2	Two ipsilateral	45 (43–48)	1:1	NCS	No	19 months–36 months
Kwon et al.^26^	2011	Observational	8/64 (12.5%) versus 4/48 on PD (8.3%)	Undisclosed	Undisclosed	Undisclosed	NCS	No	45.6 months ± 41.9 months
Warren and Otieno^27^	1975	Case report	23/36 (64%)	Undisclosed	40.8 ± 6.3	17:6	Clinical examination	No	1.8 ± 1.3 years

AVF: arteriovenous fistula; CTS: carpal tunnel syndrome; HD: haemodialysis; NCS: nerve conduction study; PD: peritoneal dialysis.

### Incidence of carpal tunnel syndrome

The frequency of CTS with AVF on HD ranged from 10.4% to 42.6% in the analysed studies.^24,26–28^ However, a study by Kwon et al.^26^ did not find a difference between the incidence of CTS in patients with AVFs (n = 57) compared with central venous catheters (n = 7, p = 0.816). More broadly, the frequency of CTS was not different in the HD group (n = 64) versus peritoneal dialysis (PD; n = 48, p = 0.823), with a mean duration on HD and PD of 45.9 ± 41.9 and 59.4 ± 44.7 months, respectively. The investigators felt that these differences could be explained by the fact that prior studies used clinical findings without nerve conduction study confirmation and recommended accurate diagnosis of CTS to be undertaken early by electrophysiological studies.^26^

### Natural history/duration of HD

Kopec found a significant correlation between duration of HD and CTS development, with all patients on ⩾20 years of HD developing CTS and requiring surgical release.^24^ In this study of 386 patients, 40 of whom who had CTS were on HD for an average of 16.05 years as opposed to the non-CTS group who were on HD for an average of 4.51 years. CTS developed in all patients dialysed from 20 to 30 years (n = 10), in 67% of patients dialysed for 15–19 years (n = 10) and in 42% of patients dialysed from 10 to14 years (n = 16). Duration of dialysis therapy was the only statistically significant risk factor for CTS.^24^ Kwon et al.^26^ also found a tendency for patients on HD to develop CTS later, although this was not statistically significant. However, only a small proportion of Kwon’s patients on HD developed CTS (n = 8) and so the study may have been underpowered, making it difficult to reach statistical significance.

### Aetiology

The development of CTS in patients on HD with AVFs may differ from idiopathic CTS in several ways. One study by Warren and Otieno^27^ showed patients with an AVF on the wrist have a significantly increased risk of developing CTS on the same side. Of 36 patients, hand volumes were investigated in symptomatic patients to find that non-dominant hands with AVFs (18%, p < 0.001 and 21%, p < 0.01 in males and females, respectively) had an increased volume compared to age-matched controls (5.1%, p > 0.1 and 5.7%, p > 0.1 in males and females, respectively). This is of importance as patients tend to have their fistulas on the non-dominant hand, while CTS tends to occur on the dominant hand in the general population.^28^ Lindstedt first reported persistent swelling of the forearm and hand in patients with iatrogenic AVFs. The authors suggested that oedema may be due to valve destruction of superficial veins distal to the AVF. This process is furthered when the arm is compressed to halt bleeding after HD and the superficial venous pressure may reach the level of systemic arterial pressure. Thus, it is postulated that this process encroaches on the crowded carpal tunnel which can compress the median nerve.^29^ This is echoed in oedema after trauma or associated fluid retention in pregnancy.^25^ Warren also found that venous pressures were significantly raised before HD,^27^ while Kocyigit found that venous pressure was significantly reduced following CTS decompression.^23^ Moreover in the latter study, amyloid deposition as stained by Congo red was absent in all of their patients biopsy specimens.^23^ Both of these studies point towards venous hypertension as a possible aetiological factor in CTS pathogenesis, however, do not elucidate on underlying mechanism for reduction in venous pressure post treatment.

β2-microglobulin amyloid deposits are another proposed aetiological factor in CTS with AVF and was found in all biopsied samples after surgical decompression in 1 study of 21 patients.^24^ β2-microglobulin deposition causes inflammation which leads to adhesions and oedema that in turn mechanically compress the median nerve, in dialysis independent of vascular access type. However, no comment was provided on the overall mean duration of dialysis in the biopsied patients. It may be that the majority of these patients with amyloid deposits had been on HD for a prolonged period of time leading to accumulation of β2-microglobulin in the tendons and synovium,^24^ and thus, symptoms of CTS may have developed regardless of presence of AVF. Importantly, Kocyigit did not find any amyloid deposits on histology of surgical specimens as discussed earlier, indicating that CTS development could not be explained by amyloid accumulation. However, the mean duration of HD for Kocyigit’s patient cohort was 3.5 years, which is considerably shorter than the mean duration of CTS patients with amyloid deposits. This may indicate that more time is required for amyloid accumulation within the carpal tunnel.^24^ It is clear that the aetiology is multi-factorial and treatment must take patient specific factors into account.

### Type/site of AV fistula

In a small case report of two patients in 1977, it was suggested that CTS was provoked by a vascular steal mechanism related to Cimino-Brescia fistula at the forearm.^20^ However, our review identified three further studies which did not find a correlation between incidence of CTS and site of AVF.^22,24,26^ Furthermore, no statistical differences were noted between the requirement of surgical procedure for CTS and location of AVF (p = NS).^24^ However, it is acknowledged that the site of fistula is not the sole aetiological factor in dialysis-related CTS as Kopec found that CTS occurred bilaterally in HD patients with AVFs, indicating that a systemic aetiology perhaps related to dialysis CTS may be involved.^24^

### Concomitant steal syndrome

In a study of 170 patients with CTS in AVF, three patients were found to have concurrently diagnosed steal syndrome. The diagnosis of vascular steal phenomenon was made by characteristic examination findings and demonstration of reduced, absent and/or retrograde digital blood flow in the distal part of the radial artery with the aid of a Doppler ultrasonographic probe.^26^

### Clinical/electrophysiological diagnosis

The symptomatology of CTS may also present a dilemma, as peripheral neuropathy, vascular steal syndrome and CTS can cause pain and/or numbness of the hands. Hence, the differentiation of the three entities is important and this involves understanding that the methods used by authors of these studies to diagnose CTS. Several studies have observed that not all methods are equal in reliably diagnosing CTS. Nevertheless, six of the eight analysed studies utilised nerve conduction studies (NCS) to aid diagnosis.^22–26^ It is important to note that uraemic peripheral neuropathy may also present in a similar fashion to CTS. Interestingly, generalised sensory and motor neuropathy was diagnosed in 12 patients (19%) as detected by neurophysiology.^26^

There is poor agreement between Hand Surgeons on the role of pre-operative neurophysiology in the management of CTS. A variety of validated neurophysiological grading systems of severity have been well-described in the literature and the Canterbury and Padua grading scales feature prominently in current UK practice in the general population.^18,29^ These have been shown to correlate well with disease severity and surgical outcomes, however, were not utilised in the studies identified. Thus, in addition to the diagnostic benefit of NCS in identifying median neuropathies across the carpal tunnel, severity and prognosis can be reliably determined.

### Steroid therapy

In a case report by Harding, only one patient had temporary relief from prednisolone injection.^23^ In a further case report, a single patient was treated conservatively with splinting with partial relief.^25^ In the authors’ practice, minimal symptoms with minor neurophysiological changes are treated with non-operative measures such as splinting, activity modification and steroid injection. In the presence of muscle wasting and sensory blunting as well as intrusive symptoms, there is little role for splinting and steroid therapy.

### Surgical management

Established CTS treated by surgical release demonstrated alleviation of symptoms and improved function of hand and quality of life in the majority of patients in the examined studies. In a cohort of 19 patients with CTS on HD (17 with AVFs), early and regular screening using NCS was suggested for early detection of CTS leading to early treatment, which can include a splint or surgical decompression.^21^ In the study, 85% (n = 16) reported improvement in symptoms, with only scar pain remaining in 21% (n = 4) at an average follow-up of 18.6 months. In a further study, two patients in a study of 36 patients with CTS underwent surgical decompression and both reported good relief and no recurrence at average follow-up of 18 months.^27^ In addition, a case series of two patients, partial relief of CTS was obtained by ligation of the AVF, whereas complete relief was obtained by surgical decompression of the median nerve at the wrist in both patients.^25^ A further study of 40 patients by Kopec found rapid relief of paraesthesia and pain symptoms. In the treatment of recurrences, resection of the thickened tendinous sheaths of the finger flexor was recommended.^24^ In a case report by Kumar, one patient was treated with surgical release with relief of paraesthesia but persistence of oedema.^25^ Surgical release for patients was required in 100% (n = 5) who dialysed for 25–30 years in a study by Kwon et al.^26^

All patients treated with surgical release in this review were reported to experience partial to complete relief suggesting that carpal tunnel decompression is a worthwhile intervention for these troubling symptoms. In this particular cohort of patients, presentation may be later on with more advanced symptoms and there is no scope here for non-operative measures. The authors’ recommendation is that carpal tunnel release should be therefore performed by an experienced hand surgeon.

### Limitations

Limitations of this study are the heterogeneity and lack of methodological detail in a relatively small sample of studies of the diagnosis and management of CTS in AVF. There are compelling reasons to identify the aetiology and best management of CTS in HD with AVF in order to assess and negate the risk of occurrence of the condition.

## Conclusion

It is clear that the tingling hand post AVF formation presents a common diagnostic conundrum for the vascular access surgeon in the setting of other differentials such as peripheral neuropathy and vascular steal syndrome. It is apparent that the aetiology remains multi-factorial and frequency of CTS with AVF varies widely according to the criteria and methods used for the diagnosis. There appears to be two distinct categories of dialysis-related CTS; venous hypertension^24^ and dialysis-related amyloidosis,^25^ although the latter findings were not reproducible.^26^ There is currently very little new evidence and a great deal of heterogeneity within the current literature. Further large scale, high-quality prospective studies are required to investigate the pathophysiology of AVF-related CTS to help improve the quality of care for HD patients. Clinicians should be aware of the diagnosis and try and make it early as possible to prevent permanent nerve damage. It is recommended that in the presence of carpal tunnel like symptoms, early neurophysiology is performed with expedited release of the carpal tunnel if symptoms and nerve tests confirm median nerve compression.
